# Pain assessment from Swedish nurses' perspective

**DOI:** 10.1111/jspn.12317

**Published:** 2020-11-02

**Authors:** Nina Skog, Mirella Mesic Mårtensson, Anna‐Karin Dykes, Vedrana Vejzovic

**Affiliations:** ^1^ Pediatric Section 1 Skåne University Hospital Malmö Sweden; ^2^ Department of Care Science, Faculty of Health and Society Malmö University Malmö Sweden

**Keywords:** nursing, pain assessment, pediatric care

## Abstract

**Methods:**

This study is a qualitative interview study. The authors used the stimulated recall interview (SRI) with nurses working at a children's hospital in southern Sweden for the data collection. In total twelve nurses were interviewed and qualitative content analysis was used for the data analysis.

**Results:**

The results are presented as one theme: Need for higher competencies and evidence, and three categories: Routines can enable pain assessment, Trusting one's own assessment of the whole picture, and Pain assessment scales as an extra workload. The interviewed nurses acknowledged that pain assessment tools are a vital part of the field of pain treatment. They also had trust issues with measuring and estimating pain by means of a tool such as pain scale. Furthermore, their opinion was that too many different tools and methods add up towards a more blurry and stress‐related environment and due to a lack of consistent routines, pain assessment is seen as a work‐related burden in the daily routines.

**Conclusion:**

Results from the present study indicated that nurses need clear routines in combination with continued education regarding pain assessment with pain scales, which might be the key to successful pediatric pain assessment and thus to better pain management within pediatrics.

## 1 INTRODUCTION

Pain relief is a human right (International Association for the Study of Pain, IASP, 1979), but the assessment of children's pain and subsequent treatment are often based on the feeling and assumptions of the nurse (Finley et al., 2005; Vejzovic et al., 2020). The knowledge regarding children's pain has increased since the 1980s, yet results from several studies show that children's pain is still undertreated (Hiller & Suominen, 2017; Lundeberg, 2014; Vejzovic et al., 2020). Although the assessment of pain has been demonstrated to have a crucial role in effective pain management, research has shown that research has shown that pain assessment using self‐reported pain is only used sporadically (Ljusegren et al., 2011; Reyers, 2003; Simons & MacDonald, 2004; Vejzovic et al., 2020). Most nurses working with children in pain still rely on their “clinical eye.” An example of this can be found in a study by Zisk‐Rony et al. (2015), who report that merely 25% of the nurses in their study used pain assessment scales when they made pain assessments. There are many factors that can make adequate pain assessment difficult, according to the result of studies based on the perceptions of nurses. Among those factors, negative attitudes, a heavy workload, the routines of the ward, and the behavior of the child, were reported as prominent in some studies (Gimbler‐Berglund et al., 2008; Manworren, 2000; Twycross & Collins, 2013). However, the main reasons for undertreated pain within pediatrics could, according to self‐reports from nurses, be a lack of knowledge and a negative workplace climate (Walker & Wagner, 2003). Ten years ago there was still a sense that it is acceptable to let children experience pain (Ljusegren et al., 2011). Nurses claim that it is necessary to be critical of the patient's experience to be able to make an adequate pain assessment, since there are, according to nurses, patients who exaggerate their pain (ibid.).

It has been shown that if many different scales are used within the same unit, this may have negative effects on the use of the scales (Finnström et al., 2008). Nurses have reported that they feel frustrated when they have access to too many scales (Zisk‐Rony et al., 2015).

Even though research has been able to show the importance of pain assessment, adequate recommendations for both acute and chronic pain are still missing within Swedish health care (Alfvén et al., 2012). A recent study by Vejzovic et al. (2020) has also shown that children as inpatients still experience pain during hospitalization. When inpatient children from four countries in Europe were asked to self‐report if they had pain, 87% (503/579) reported pain during the past 24 h. Since children still suffer from pain during their hospitalizations, there is a need to understand why. One of the ways to increase understanding was to investigate the nurse's experiences of working with pediatric pain assessment. As pain assessment is the essential first step in pain management, there is a need to understand which pain assessment methods are used and why nurses choose them. The purpose of this study was thus to increase the understanding of Swedish nurses' views on the assessment of children's pain, with a focus on pain scales.

## 2 METHODS

The present study has a qualitative design, where the stimulated recall interview (SRI) technique (Bloom, 1953; Lyle, 2003) was used for the data collection, and qualitative content analysis Graneheim et al., 2017; (Graneheim & Lundman, 2004) was used for the data analysis. A written permission to conduct the study was provided by the clinic manager at a children's wards university hospital in the south of Sweden. The study was performed in compliance with the ethical guidelines of the Declaration of Helsinki (WMA, 2013). Written information about the study, including that participation was voluntary, was provided to the nurse's before recruitment. Furthermore, written information in the shape of a poster was published at the clinic, and nurses were asked to participate in the study and were also asked to assist researchers in identifying other potential research subjects, to obtain a snowball effect.

### 2.1 Data collection

The interviews, conducted during March and April 2019, were done using the SRI technique. The participants could choose the time and place for the interview, which was conducted during their working hours. Each informant signed an informed consent form before their interview was initiated. All interviews were recorded by means of two sound recorders to minimize the risk of losing recorded material. The interviews lasted between 16 and 26 min (median 19.31 min). The central idea behind SRIs is to use a stimulus, usually in the shape of video‐recorded or sound‐recorded material (Lyle, 2003), or written patient cases (Liimatainen et al., 2001). SRIs are used in situations where we want to give rise to and extract thoughts and/or ideas (Bloom, 1953) or elicit a more detailed narrative about how and why certain decisions should be made (Sinnot et al., 2017). The stimulus used in this study was a real‐life patient case reflecting a situation that most nurses who work with children have experienced. The written patient case was read by the informants in connection with the interview. Thereafter, the SRI started with an open question: “Now that you have read about the case, could you tell us about your thoughts with regard to caring for this patient?” Short follow‐up questions, such as “Could you elaborate on this?” and “How do you reason?,” were formulated to clarify the descriptions of the informants. After 12 interviews with nurses with varying professional experience, data saturation was reached and the amount of data was considered sufficient for the researchers to be able to see similarities and differences in the material. The collected data were not only considered sufficient but also manageable enough not to lose sight of the overall picture.

### 2.2 Data analysis

The data were analyzed using qualitative content analysis both at the manifest and the latent level, according to Graneheim and Lundman (2004). Following Graneheim et al. (2017), a descriptive theme was used to describe the major thread of the data and answer the question: What are these nurses trying to tell us? After each interview, the interviewers summarized the conversations and received an oral confirmation from the informant that everything that had been said was perceived correctly. The recorded interviews were transcribed verbatim. Then the texts were read through individually in order for all authors to familiarize themselves with the text and get an overall picture of the material. In the next step, meaning units corresponding to the aim of the study were identified and compared. Authors N. S. and M. M. M. first created meaning units individually and then compared the units with each other until agreement was reached. Each meaning unit was then coded, and to reduce the risk of altering the meaning, the authors returned to the material several times by listening to the recordings and reading the transcribed material. The codes were distributed according to similarities and differences in relation to the aim, resulting in one theme and three main categories. Categorization can be a critical element in the analysis process, and since the categories must be complete and mutually exclusive, we chose to create subcategories that would then lead us to categories. This was done to facilitate the process and so that all meaningful units should belong only to a relevant category. The authors have collaborated a great deal to be able to reason about and analyze the material, which increases the trustworthiness of the result. One potential risk for the misinterpretation of data was that N. S. and M. M. M. have a preunderstanding, since they work clinically with children suffering from pain. To minimize this risk, the awareness of their pre‐understanding was repeatedly discussed and reflected on together with A. C. D. and V. V. This reflexivity, in combination with discussions and interpretations of all the findings by authors with different professional backgrounds, may have further contributed to the study's credibility. Examples of manifest analysis are presented in Table 1.

**Table 1:** The sample characteristics

Meaning unit	Condensed meaning unit	Code	Subcategory	Category
“It hurts, yes, it does. But if you want to get a completely clinical view and the clinical state of the patient, it's not just the pain that you look at, but the whole picture, together with the background, the reason for the hospitalization, that is.” Informant 2	Trusts the clinical eye in combination with the overall assessment.	Trusts the clinical assessment of the whole picture		Trusting one's own assessment of the whole picture
“There are of course a number of suspicions around it. You think the scales aren't good and you think they are misleading or something, And then it's also a matter of lacking time and such.” Informant 11	Suspicions regarding the scales exist; they are considered misleading.	Misleading working tool.	There are many scales to choose between.	Pain assessment scales as an extra workload.
			Trust in scales is weak.	

## 3 RESULTS

The results of this study are presented as one theme: Need for higher competencies and evidence, and three categories: Routines can enable pain assessment, Trusting one's own assessment of the whole picture, and Pain assessment scales as an extra workload.

In total, 12 nurses, who worked in three different children's wards in a hospital in southern Sweden, were included. The inclusion criteria were: registered nurses with at least 1 year's work experience within pediatrics. The work experience of the informants varied between 1 and 30 years (median 6.5 years). Ten of them were registered nurses (RN) with specialist education within child and adolescent care and two of them were RN. All of them were women. Their work experience in pediatric care is presented in Table 2.

**Table 2:** Examples of categorization

Informants	Work experience in pediatrics	Examination as a registred nurse	Examination as a specialist nurse
1	Since 2013—5 years	2010	2012
2	Since 2011—7 years	2006	2010
3	Since 2016—3 years	2016	–
4	Since 2017—2 years	2013	2017
5	Since 2018—1 year	2012	2018
6	Since 2014—5 years	2013	2019
7	Since 2018—1 year	2015	–
8	Since 2012—7 years	2012	2018
9	Since 2002—17 years	1993	2012
10	Since 2008—11 years	2007	2011
11	Since 1990—29 years	2008	2014
12	Since 2012—7 years	2012	2016

### 3.1 Need for higher competencies and evidence

This theme emphasized the competence in pain assessment that pediatric nurses need to help children who experience pain as inpatients. It is imperative that nurses who work with children have enough competence and that they use evidence‐based methods when they assess pain. The nurses in this study did not feel comfortable with pain assessment, especially when they should base it on the use of a pain scale. According to pediatric nurses' experiences, up‐to‐date knowledge is necessary in pediatric care. Studying pediatric nurses' experience of pain assessment showed a need to strengthen their opportunities for increased competence and to help them to use evidence when they need it. The nurses thought that clear routines for the assessment of pain in children might have a positive effect. It emerged that established routines exist within surgical care and that the nurses found the pain assessment taking place there to be well functioning. Pain assessment was described as a natural part of surgical care. The nurses who had experience of working with postoperative pain saw the pain assessment as a good routine, where regular documentation as well as the evaluation of the effect of the treatment are included.

### 3.2 Routines can enable pain assessment

The nurses saw pain assessment as an important part of pain management. However, they experienced a lack of routines when they needed to use different instruments for the assessment. Asking both the accompanying parent and the child about pain, was natural and important, but using a pain scale was not as self‐evident. Routines regarding pain assessment were not clear for nurses. The scales mentioned varied; both self‐assessment scales and observation scales were referred to.

The nurses reported that the understanding of pain assessment methods may affect the possibilities for developing one's competence in the field.

Above all, education is needed in order to become better at pain assessment, and that everybody does it./…/ It's just like measuring the pulse rate, the blood pressure, check‐ups, and fever. Making this part of one's work, actually, that's how important it is, and that it becomes a routine.

(Informant 11)

Furthermore, the nurses felt that they assessed children's pain more often if the scales were easy to access, for example, by hanging in the patients' rooms, where the nurses were reminded of the scale and where it also gave the children the possibility to acquaint themselves with it.

/…/ You see them when you go in to fetch them and use [them]. /…/ And that the patients themselves can see them and wonder about what they are and look at them and perhaps bring them up themselves.

(Informant 6)

### 3.3 Trusting one's own assessment of the whole picture

From the narratives, it emerged that the anamnesis as well as the child's pain signals were included in the assessment of the child's pain. Through the anamnesis, the whole picture of children's pain was sought. The nurses stressed the importance of getting an overall picture of the child's situation to be able to assess and evaluate her/his pain. Pain assessment instruments were seen as merely complementary to the holistic assessment, as they were not, by the nurses, considered to give sufficient information about children's pain. When nurses assessed the pain without using the scales, they relied on their own “clinical eye,” which was described as being able to trust one's own feeling based on knowledge and experience. Nurses' perception of the concept of the clinical eye was described as the observation of the child's appearance, behavioral changes, and body movements.

Children can usually not counterfeit not being in pain when they are, but if they walk with a straight back or a little hunched when they don't think that you are watching. Some body language. How they sit in bed. If they are in great pain but still sit with their legs crossed then perhaps they aren't in so much pain when they can sit like that or stretch them out, they put less weight on that body part. Movement patterns above all. If they can, sort of, do deep breathing.

(Informant 1)

The nurses described how the clinical eye improved with experience. One's own experience of being in pain might play a crucial role in pain assessment, they said. All experience was counted, such as experience of working with pain, that is, experience acquired through professional practice, and experience acquired through education. They felt that experience is required to assess and observe pain signals, and described this as something that is difficult in the beginning of one's career.

I remember my first patient with a disability /…/ my boss at the time was with me because I was so new /…/ and she told me that he's in much pain and I didn't get what she meant because I didn't see it at all. He was lying with his hands clenched into fists and just totally quiet, totally quiet he was.

(Informant 8)

Experience and practice are needed also to be able to use pain assessment scales. The nurses described it as being “much more difficult to make a pain assessment when you don't have any experience. Even if you have an instrument for it, it's just a part of the picture” (Informant 3). However, in the beginning of the nursing career, before having been able to develop one's clinical eye as a nurse, the pain assessment scales were perceived as a good support in becoming aware of children's pain.

### 3.4 Pain assessment scales as an extra workload

Trust in the pain assessment instruments was felt to be low among nurses. It emerged that there were many scales for them to use, which resulted in the nurses sometimes considering pain assessment an extra workload. When nurses experienced pain assessment scales as an extra workload, the use of them was deprioritized. Many different reasons were mentioned for the nurses' choice to deprioritize the use of pain assessment instruments. Reasons described were lack of time, stress, and organizational deficiencies. “/…/ you shouldn't always blame it on a lack of time… but I also think it's part of it” (Informant 12). Periodically, when there was a lot to do in the workplace, the nurses felt stressed and correct pain assessment was not performed. Moreover, the fact that pain assessment instruments were deprioritized was sometimes felt to be due to the culture in the workplace.

It's sort of deeply rooted in the culture here that we don't remind each other /…/We don't make pain assessments in this ward.

(Informant 10)

If the pain assessment scales were not a clear part of the work, then pain assessment instruments were not used but instead experienced as an extra workload.

I actually think that it's deprioritized by most of us /…/ Am I good at using pain scales? No. Pain assessment isn't made. I know full well that it's not good. I can say that sometimes it's due to laziness, I don't know. I deprioritize it. I usually don't think about it.

(Informant 7)

It was considered important that the working group have a positive attitude to pain assessment. Nurses reminding each other and realizing the importance of making pain assessments with the help of an instrument, was perceived as something that could increase the use of pain assessment scales.

#### 3.4.1 When there are many scales to choose between

The nurses said that currently there are many scales they can use within pediatrics, and because of the great number of pain measurement instruments the nurses sometimes felt that it was difficult to choose. That could be one reason why the scales were not used.

It may be that we have too many tools, it's easily deprioritized, unfortunately.

(Informant 12)

There is a risk that the assessment will not be objective and that it will be hard to compare the assessments over time, if several different scales are used for the same child. The nurses described it as confusing both for the child and for the nurses to use several different scales, as the child, for example, does not have time to acquaint herself/himself with the scale.

/…/ But being on the same track matters a lot of course, and the patient not getting a new scale every time and being confused.

(Informant 4)

When there were many scales to choose between, the nurses felt that they were not always told what scales their colleagues used, as the choice of pain assessment scale was not always reported. The transfer of information on the ward could be affected and this was seen as leading to the children's pain being harder to assess. Pain was often documented descriptively in writing, without any figures, which also complicated the assessment of pain.

/…/ we sort of don't use the scale itself but we rather describe how the child seems to be doing, because it's not really implemented among us. If I had written ALPS 4 not many persons would have understood, no, so that's something we can become better at.

(Informant 1)

The nurses felt that being educated in how to use the scales would have facilitated their choice of the right scale. When there were too many scales on the wards, this was seen as a risk for the child, as children's pain could then be assessed differently. The risk arises, they said, when the assessment criteria of the scales are different:

/…/ some go from 0 to 3 and some from 0 to 5, so that you don't really learn what is a 3/…/ or a 4 according to me /…/. Instead you have different scales so that you have to sit and read what it says under a 3. It's harder to learn.

(Informant 1)

Many nurses expressed that their clinical work would have been facilitated if there had been clearer guidelines about which pain assessment scales to use.

#### 3.4.2 Trust in the scales is weak

The nurses in our study sometimes experienced limited trust in the pain assessment scales, and the result of the pain assessment was sometimes felt to be a guess or a coincidence. When the child's behavior did not correspond to the result of the pain assessment instrument, the nurses found it hard to trust that the scale really measured what it was intended to measure.

I'm a little skeptical of the numerical scale /…/ so I always add a description. When appropriate. When reasonable. The patient is watching TV or looking tense.

(Informant 2)

The nurses perceived the pain assessment scales as misleading, as there are sometimes other things than pain that give points on the scale. This could be, for example, nausea, hunger, worry, fear, or the child's previous experiences of pain. It led to uncertainty about whether the scale measured what it was supposed to measure. The hospital environment can be seen as stressful or worrying for the sick child, who can then experience pain as more intense, which will give higher points on the scale. It can be difficult for the nurse to know whether the increase in stress is related to pain. The reliability of observation scales, such as the Face Pain Scale, in particular, was questioned, as they are based solely on the observation of the nurse and do not take the child's perspective into account. The Face Pain Scale was also considered to be difficult to interpret.

The faces of the Face Pain Scale look a bit scary. I find it difficult to see the different expressions. I have tried to use it but it's not something that I'm comfortable with.

(Informant 1)

The nurses experienced uncertainty when using a scale for children with communication difficulties or multiple disabilities. “/…/ there I would never consider using NRS or any other numerical scale because it doesn't work. It's all my own perception” (Informant 6). This was due to, for example, fear of not understanding the pain signals of the child and an inability to communicate with the child. In these situations, the nurse relied on the parents. The parents were seen as an asset, as they know their child and were felt to act as an information bridge. The parents were often involved in the pain assessment and involving them was considered valuable and sometimes necessary, as the sick child cannot always assess her/his own pain.

## 4 DISCUSSION

The aim of the study was to increase the understanding of the nurses' view of the use of pain assessment scales in assessing pain. The purpose of the used method was to expand the nurses' descriptions of their experiences regarding pain assessment and its methods by using a reality‐based case. The results from the present study provided an up‐to date insight into nurses' assessment of pain in children.

Nurses in this study self‐reported their own perception of having theoretical knowledge about pain assessment of children in pain. According to the competence description for specialist nurses by The Swedish Society of Nursing (2016), a specialist nurse should be able and knowledgeable enough to actively follow research and conduct evidence‐based care within pediatrics. However, nurses in this study reported that there was a discrepancy between their theoretical knowledge and the practical exercise of it; despite the fact that the nurses found it valuable to use pain assessment scales, they are not widely used in pediatric care.

The result also showed that the nurses experienced a lack of unified and clear guidelines for assessing children's pain by means of pain assessment scales and that these were therefore deprioritized. Similar conclusions have been reported previously (Enskär et al., 2007; Finley et al., 2005; Gimbler‐Berglund et al., 2008; Pölkki et al., 2010). In the present study, self‐reported experiences varied between surgical and medical contexts. A perception seems to exist that routines and guidelines are clearer within the surgical context, something which is seen as promotive of nurses' work, compared to the medical context. However, Twycross and Finley (2013), after an observation study, reported that nurses, despite having guidelines when managing postoperative pain, did not always undertake pain assessments, and that, when recorded, these were not always used to guide decision‐making. Gimbler‐Berglund et al. (2008) have demonstrated that nurses' perceptions of, attitudes to, and view of pain assessment, may affect practices. The fact that this can be problematic is shown in the result from a study by Ljusegren et al. (2011), where it emerged that nurses underestimate the child's pain compared to the child's own assessment and that this can lead to children's pain being undertreated (De Tovar et al., 2010; Khin Hla et al., 2014; Zhou et al., 2008). Different pain scales are also said to constitute a possible difficulty, as there is, according to nurses in this study, a lack of recommendations for a unified use of pain assessment scales. These problems have already been highlighted in different contexts, which confirms our results (Finnström et al., 2008; Zisk‐Rony et al., 2015). Zisk‐Rony et al. (2015) questioned the nurses' choice not to use pain assessment scales, considering that their assignment was to offer patients the best possible care. One reason for questioning the nurses' choice not to make multidimensional pain assessments is that there are children in hospital with underestimated pain (Vejzovic et al., 2020).

Clearer routines and a limitation of the number of pain assessment scales could have facilitated the nurses' choice of scale in their daily work with sick children. Egan and Cornally (2013) also stressed that the lack of organizational structure is a major obstacle to successful pain assessment. This could be important to consider in making future improvements, as results from this study indicate that the nurses, regardless of their perception of being able to process knowledge about pain, need more regular training and education within this field. Ramira et al. (2016) reported improvements after an intervention where the nurses were given continuing education about pain assessment scales. To fulfill the requirements set by the UN Convention on the Rights of the Child (1989) and minimize the risk of gaps in the care given to children, we need to convert theoretical knowledge into practical work. The recommendation is to start from the child's perspective, which in this case is the child's experience of pain, and therefore it cannot be accepted that pain assessment is made without involving the children concerned. Previous research indicates that there is still a great deal of work to be done do to reduce the gap between theory and practice, and we believe that it is time to include pain assessment training based on both international and national guidelines as an intervention, to try to reduce this discrepancy.

The results from this study show that despite extensive research on the importance of using pain scales regularly in pain assessments, there are still shortcomings in this area in pediatrics, according to nurses interviewed for this study. Furthermore, the current study has been able to confirm previous research showing that a lack of clear routines and of further training in the area may be reasons for the shortcomings in pain assessment, something which may in turn have a negative impact on pain treatment (Nilsson, 2016; Ramira et al., 2016).

None of the existing scales are adapted to all ages (Tsze et al., 2013) and therefore there is still a need to develop pain assessment scales, especially for younger children (Von Baeyer et al., 2017). The pain assessment scales are constantly evolving; there is, for example, a revised version of FLACC, r‐FLCC, but it has not been validated in a Swedish context (Nilsson, 2016; Voepel‐Lewis et al., 2008). In pediatrics that includes children of different ages (in Sweden 0–18 years) and requirements for self‐assessment of pain as far as possible, the challenge for nurses will continue to exist, but with more education and regular use of selected pain scales, the work with pain assessments can be facilitated and reduce these challenges.

### 4.1 Strengths and limitations

The interviews in this study were done by means of the SRI technique, which can be seen as a strength because it increased the possibilities for getting detailed narratives about the nurses' procedures in the kind of situation described in the real‐life case. The SRI technique was also a way of opening up for discussion as well as easing the atmosphere, as the informants had a concrete situation to relate to. The weakness of using SRI is that the method is not sufficiently tested and established in care science research (Sinnot et al., 2017).

Since the authors wanted to obtain their results from nurses working in general pediatric, neonatal, intensive care, postoperative wards were excluded. It cannot be ruled out that the results might have been different if the selection had included the latter wards. If it had been possible to perform a more extensive study, it would have been valuable to include those units as well.

## 5 CONCLUSION

The present study aimed to increase the understanding of Swedish nurses' views on the assessment of children's pain, with a focus on pain scales. Results from the present study indicated that nurses need clear routines in combination with continuing education regarding pain assessment with pain scales, which might be the key to successful pediatric pain assessment and thus better pain management within pediatrics.

## What is currently known?

The knowledge regarding children's pain has increased since the 1980s, yet results from several studies show that children's pain is still undertreated. Pain assessment is the essential first step in pain treatment; however, it can be challenging in a pediatric context due to a variety of factors, including age (0–18 years) and different pain scales that arerecommended for pain assessment (Hiller & Suominen, 2017; Lundeberg, 2014; Vejzovic et al. 2020). Self‐report of pain is an important part in pain assessment, and there are a variety of pain scales available in pediatric care. Previous research results have shown that nurses consider that they have knowledge about them and that they are aware of the importance of using them when assessing pediatric pain.

## What does this article add?

The purpose of this study was thus to increase the understanding of Swedish nurses' views on the assessment of children's pain, with a focus on pain scales. This paper adds new insight into how the nurses experience pain assessment and why they use pain scales sparingly. Results from this study support and correlate with previous research that promotes pain assessment as an important part of pain management. Moreover, the result of this study offers new understandings, which can contribute to the standardization of pain assessment methods and which may benefit practice regarding pain assessment in pediatrics.

## CONFLICT OF INTERESTS

The authors declare that there are no conflict of interests.

## AUTHOR CONTRIBUTIONS

N. S., M. M. M. and V. V. was responsible for study concept and design. N. S. and M. M. M. was responsible for data collection. N. S., M. M. M., A. K. D. and V. V. was responsible for data analysis and interpretation. N. S., M. M. M., A. K. D. and V. V. was responsible for drafting of the article.
